# Supplementary material for: The Association Between Prevotella copri and Advanced Fibrosis in the Progression of Metabolic Dysfunction-Associated Steatotic Liver Disease
Source: Nutrients. 2025 Jun 27;17(13):2145. doi: 10.3390/nu17132145 (PMC12251637; doi:10.3390/nu17132145)
Supplement: Supplementary file 1 [file nutrients-17-02145-s001.zip › nutrients-3720968-supplementary.pdf]

Supplemental Table S1: Primer sequence

Gene	Forward (5′-3′)	Reverse (5′-3′)
Cpt1	TGT CCA AGT ATC TGG CAG TCG	CAT AGC CGT CAT CAG CAA CC
Dgat	GGC CTG CCC CAT GCG TGA TTA T	CCC CAC TGA CCT TCT TCC CTG TAG A
Atgl	GGT CCT CTG CAT CCC TCC TT	CTG TCC TGA GGG AGA TGT C
Ocln	CTC GAG AAA GTG CTG AGT GCC TGG AC	AAG CTT TCG GTG ACC AAT TCA CCT GA
Zo	GGG AGG GTC AAA TGA AGA CA	GGC ATT CCT GCT GGT TAC AT
Cldn	CTC GAG GGA AAC TAC AGT CCC AGC GA	AAG CTT GAT GTT GTC GCC GGC ATA GG
